# Biocatalysis for Biobased Chemicals

**DOI:** 10.3390/biom3040812

**Published:** 2013-10-17

**Authors:** Rubén de Regil, Georgina Sandoval

**Affiliations:** Unidad de Biotecnología Industrial, CIATEJ, A.C. Av. Normalistas 800, Col. Colinas de la Normal, Guadalajara, Jal, C.P. 44270, Mexico; E-Mail: ruben_de_regil@yahoo.com

**Keywords:** biocatalysis, hydrolases, transferases, polymers, biodiesel, fine chemicals, prebiotics

## Abstract

The design and development of greener processes that are safe and friendly is an irreversible trend that is driven by sustainable and economic issues. The use of Biocatalysis as part of a manufacturing process fits well in this trend as enzymes are themselves biodegradable, require mild conditions to work and are highly specific and well suited to carry out complex reactions in a simple way. The growth of computational capabilities in the last decades has allowed Biocatalysis to develop sophisticated tools to understand better enzymatic phenomena and to have the power to control not only process conditions but also the enzyme’s own nature. Nowadays, Biocatalysis is behind some important products in the pharmaceutical, cosmetic, food and bulk chemicals industry. In this review we want to present some of the most representative examples of industrial chemicals produced *in vitro* through enzymatic catalysis.

## 1. Introduction

It is becoming evident that in order to sustain the standard way of life of the developed and in-development world, it will be necessary to make some adjustments either to our consumption habits or to our sources of supplies of energy and materials. In the latter case, Biotechnology, as a diversified discipline in which chemistry, physics, biology, optics, electro-magnetism and thermodynamics converge, possess the knowledge tools to play a relevant and vital role to address the challenges of growth, ageing, employment, limited sources of raw materials, energy and water supply and living standard. Industrial biobased processes will increasingly become the *de facto* alternative to build a sustainable economy. It is estimated that, by 2030, the products of white biotechnology and bioenergy will account for 30% of industrial production worth €300 bn [1].

Conversion of biomass by Biocatalysis is likely to become standard technology which will contribute significantly to open up access to large feedstock supplies for bioprocesses and the production of transport fuels. On the health and ageing field, personalized nutrition, tailor-made medicine will become a reality in the coming years thanks to novel, specific biotech drugs and regenerative medicine obtained by more efficient and greener processes.

Biocatalysis refers to the transformation of substances of chemical or biological origin through the use of the enzymes produced by diverse living organisms. Enzymes may carry out reactions in a free, or immobilized form, or within the living cell in which they naturally live.

Isolated enzymes obtained as purified and concentrated extracts, whether used immobilized onto a support or in free form, may produce neater products than when the whole microorganism cell is used for the biotransformation. This is because of the absence of other kinds of enzymes from the internal cell machinery, which may subsequently modify the product, and the absence of internal cellular components during the lysis-extraction step. This advantage of isolated enzymes has a positive impact on yield and purification costs. However, isolated enzymes are known to be less stable in a purified form than within the cell. The lower stability of free enzymes has been improved to some extent by immobilization and crosslinking techniques, the results of which vary depending on the type of enzyme, pH memory, extent of crosslinking, immobilization support and the procedure itself.

Whole microorganism cells, obtained in sufficient amount by fermentation, are used instead of isolated enzymes when their enzymes become highly unstable or non-functional outside the inner environment of the cell, so catalysis is confined within the cell and products are later extracted from it. 

Chemical synthesis in the food, feed, industrial and pharma sectors is the field to which Biocatalysis has contributed the most, but it has also contributed to the bioremediation sector, another field in which enzymes are having a significant role.

The relative success of Biocatalysis applied to Synthetic Chemistry has been due mainly to the high enantio- and regioselectivities that enzymes exhibit towards their substrates, because enzymes speed up chemical processes that would otherwise run very slowly or without selectivity, and because enzymes function under mild reaction conditions. These advantages have allowed chemists to avoid the burden of group-protecting procedures, saving time, materials (including the harsh, dangerous or toxic ones) and energy costs. Other advantages of enzymes are that they are easy to control and biodegradable. Thus, Biocatalysis has proved in many cases to be a superior pathway than conventional chemical synthesis pathways, not only in the simplicity of accomplishing the reactions, but also from an economical and environmental point of view.

An important landmark in Biocatalysis was the use of organic solvents as reaction media, as it was thought that enzymes may only work in aqueous medium, naturally. Organic solvents became an important aid in dissolving organic, hydrophobic molecules, which account for much of the library of organic chemicals used in Synthetic Chemistry. The possibility to carry out reactions in organic media favored the reactions rate precisely because of the better solubility of reactants. Purification steps became shorter and easier because the avoidance of surfactants needed in aqueous media and the use of low boiling point solvents easy to evaporate. Moreover, hydrolytic, secondary, water-dependent reactions responsible for lower product yields, and growth of microbes in containers and pipes, is avoided because of the absence of water and the harsh effects of solvents to living cells. 

However, Biocatalysis still needs to overcome some limitations such as the enzyme costs of production and the relative narrow stability window of many enzymes under diverse reaction conditions, which restricts their applications onto broader industrial processes. Chemicals used in industrial settings are mostly derived from fossil oil, whereas active ingredients in pharmaceutical processes may be new and/or structurally complex. In general, a vast number of chemicals used in industry are artificial, new man-made chemicals for which enzymes did not evolve to work with and thus are not suitable for the synthetic needs in industry. So there are high discrepancies between the enzyme´s function in nature and the functions needed in the transformation industry. High throughput enzyme screening and protein engineering techniques, particularly Directed Evolution, are some of the tools being employed to advance faster in this aim to find enzymes for artificial substrates, and to create enzymes with new capabilities. 

In this review we want to present some of the most representative examples of industrial chemicals produced *in vitro* through enzymatic catalysis, and hence, *in vivo* fermentation products are not included.

## 2. Enzymes in Food Industry

Applications of enzymes in the food industry are found in almost every sector of this industry: confectionary and sweeteners, fruit and vegetables, dairy, brewery and beverages, meat, dietary and the nutraceuticals niche [2]. However, most of these applications are of hydrolytic nature focused in debranching, improving solubility, clarification, and hydrolysis aimed at diverse goals depending on the food being treated. 

### 2.1. Prebiotics and Sweeteners

**Figure 1:**
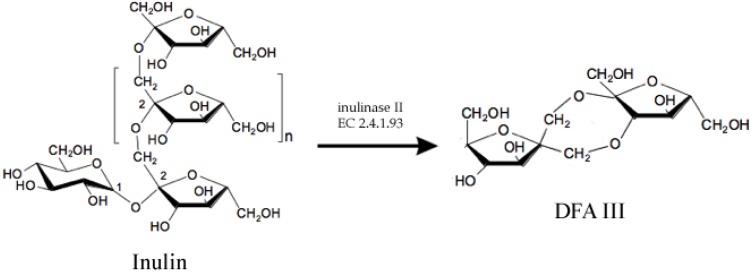
Enzymatic synthesis of Difructose Anhydride III (DFA III) from Inulin using enzyme inulase II, an inulin fructo-transferase.

There are some examples of food chemicals being synthesized by enzymes. One of them is Disaccharide Difructose Anhydride III (DFA III), which is a non-reducing, non-cariogenic sweetener with probiotic properties [3]. DFA III can be synthesized by enzyme inulase II (EC 2.4.1.93), (Figure 1), which was first identified in *Arthrobacter ureafaciens* [4] and later in other bacteria [5,6,7]. However, native enzymes are not thermotolerant enough to resist high temperatures required for a large scale process. Thus, the *ift-*gene encoding for the inulase II from Buo141, a thermotolerant strain, was cloned and expressed into *E. coli* XL1-blue and further modified and immobilized to obtain the biocatalyst which was reported to have 1.7 × 10^6^ U/L [3]. 

Prebiotics is another field in which enzymes are being used. Oligosaccharides are non-digestible saccharide polymers containing 3–10 monomeric sugar units that are found in low amounts in human milk [8,9], onion, garlic, banana, soybean and chicory [10]. They are classified as prebiotics because they selectively promote the growth of bifidobacteria and lactobacilli in the intestine, which are regarded to have a beneficial effect to human health [10,11,12,13,14]. 

Oligosaccharides composed either of fructose or galactose units are named fructo-oligosaccharides (FOS) and galacto-oligosaccharides (GOS) respectively. 

**Figure 2:**
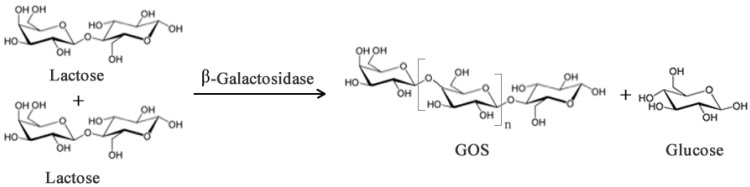
Simplified enzymatic reaction to produce Galacto-oligosaccharides (GOS) from lactose using β-galactosidase. Subscript *n* may range from 0–6 though most native enzymes produce GOS with *n* between 1–2.

Galacto-oligosaccharides (GOS) can be synthesized from lactose when this sugar acts as the acceptor and transgalactosylation is catalyzed by β-galactosidase (Figure 2). In addition to the prebiotic activity, GOS have also been reported to contribute to (i) reduction of serum cholesterol and lipid level; (ii) synthesis of B-complex vitamins; (iii) enhance absorption of dietary calcium [14,15,16]; (iv) protection from infection and decrease of pathogenic bacteria; (v) stimulate absorption of some minerals [17]. However, these health promoting properties vary depending on the chemical composition, structure and degree of polymerization and these features depend, in turn, on the origin of the β-galactosidase [18].

The most recurring means of the synthesis of GOS is by enzymatic catalysis from lactose using glycosyltransferases (EC 2.4) or glycoside hydrolases (EC 3.2.1) [19]. Glycosyltransferases are not used in the synthesis of GOS due to their low availability, the need for nucleotide sugar substrates and prohibitive prices. Instead, glycoside hydrolases are used, despite the fact that they are less stereoselective than the former [20]. Glycoside hydrolases from archeas (*Sulfolobus solfactaricum* and *Pyrococcus furiosus*), bacteria (*Saccharopolyspora* sp., *Bifidobacterium* sp., *Thermotoga* sp., *Thermus* sp., *Bacillus* sp., *Geobacillus* sp., *Caldicellulosiruptor sp.*, *Lactobacillus* sp., *Streptococcus* sp., *Enterobacter* sp., *Escherichia* sp.) and yeasts (*Aspergillus* sp., *Penicillinum* sp., *Talaromyces* sp., *Trichoderma* sp., *Kluyveromyces* sp., *Sirobasidium* sp., *Sterigmatomyces* sp., *Rhodotorula* sp., *Sporobolomyces* sp., *Rhizopus* sp.) have been tested for the synthesis of GOS [19,21].

Milk whey is a lactose-rich source which has been regarded in the past as a waste by-product from cheese production highly contaminant. So β-galactosidases have become a valued natural tool to produce a beneficial product out from a contaminant waste in a single step.

As a general finding, reports show that GOS yields average 30%–35% of total sugars [22,23] and are directly proportional to the initial lactose concentration. The major product is the trisaccharide accounting for as much as 80% of total GOS synthesized [12,24]. It was estimated that at least 3,500 tons of GOS are enzymatically synthesized from whey lactose, out of 30,000 tons of total world annual production [25]. They are commercialized to enrich mainly infant formulas. 

Just like GOS, Fructo-oligosaccharides (FOS) have low caloric value and have much the same prebiotic properties due to their indigestibility in the upper gastrointestinal tract: they are non-cariogenic and have ability to promote the activity of beneficial colonic lactic acid bacteria and to modulate the intestinal immune response [26,27,28]. However, unlike GOS, FOS are naturally occurring sugars biosynthesized by numerous plants such as asparagus, sugar beet, onion, artichoke, *etc*. [29,30]. Two main types of FOS can be found in nature, the inulin type, which are fructose polymers with linkages β(2–1) biosynthesized by inulosucrases (E.C. 2.4.1.9), and the levan type, which are fructose polymers with linkages β(2–6) biosynthesized by levansucrases (E.C. 2.4.1.10) [31,32,33].

**Figure 3:**
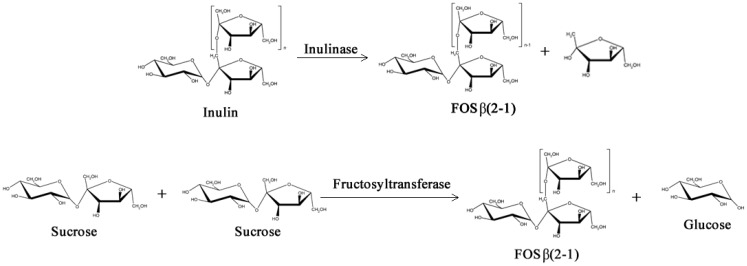
Simplified enzymatic reactions used commercially to obtain Fructo-oligosaccharides (FOS).

Two different classes of FOS mixtures are produced commercially, one is based on inulin by controlled enzymatic hydrolysis, and the other is based on sucrose by a transfructosylation processes with fructosyltransferases, as shown in Figure 3 (β-fructofuranosidase, E.C. 3.2.1.26 or β-d-fructosyltransferase, EC 2.4.1.9) [34,35]. FOS from inulin hydrolysis contain longer fructose chains (Degree of Polymerization, DP 2–9) and may have either a fructose or glucose unit at the end of the chain. On the other hand, FOS from sucrose transfructosylation contain shorter polymer chains (DP 2–4) and always end with a glucose unit [35].

A commercial enzyme Pectinex Ultra SP-L was used immobilized onto Eupergit C to synthesize FOS from a 600 g/L of sucrose solution. Reactions were carried out at 65 °C in pH 6.5 during 24 h with a yield of 57% FOS of total sugars [36].

Despite the big production numbers that prebiotics have, the overall European market for prebiotics is still at the beginning stage, with the $87-million fructan (inulin and fructo-oligosaccharide) segment as the most developed part. In US the prebiotics market is estimated to reach US$198.3 million in 2014 [37].

### 2.2. Structured Lipids

Specialty fats and oils are lipids with special functional or nutritional effects in the human body. Among these, structured lipids (SLs) have a predominant importance within this field [38,39]. SLs are tailor-made fats and oils which incorporate specific new fatty acids to triacylglycerols, or have different composition and positional distribution of existing fatty acids, within its glycerol backbone. Among the various types of SLs, the MLM (medium, long, medium fatty acid chain length esterified at the *sn*-1, *sn*-2 and *sn*-3 positions of the glycerol backbone respectively) have been a subject of great interest because of their ability to provide quick energy through the pancreatic lipase hydrolytic release of the medium chain fatty acids which become rapidly oxidized by the liver [40]. The remaining long chain *sn*-2-monoacylglyceride is left to be absorbed through the lymphatic system [41]. This long chain monoacylglyceride can be an omega-6 fatty acid or any other poly unsaturated fatty acid (PUFA) which will provide the already known health benefits to the body [42]. 

SLs are claimed to have a modulation effect on the immune system, to improve the lipid clearance in the blood [43], and as a fat for special nutritional feeding [44]. Fatty acids at the *sn*-2 position are more readily absorbed *in vivo* than those at the *sn*-1 or 3 positions, thus, SLs of the type MLM that include an essential fatty acid in the *sn*-2 position are desirable nutrition sources for people who suffer from malabsorption or a pancreatic condition [44,45]. 

SLs can be obtained either chemically or enzymatically using lipases [42]. The high regioselectivity of lipases makes Biocatalysis particularly well suited for the synthesis of SLs, avoiding the synthesis of unwanted by-products, which lower the yields [46]. However, enzymatic synthesis of SLs has yet to be optimized in order to avoid the high costs of large-scale purification of unreacted substrates (Free fatty acids, Triacyglicerides, Diacylgricerides, Esters) which must be removed upon completion of reaction [47].

**Figure 4:**
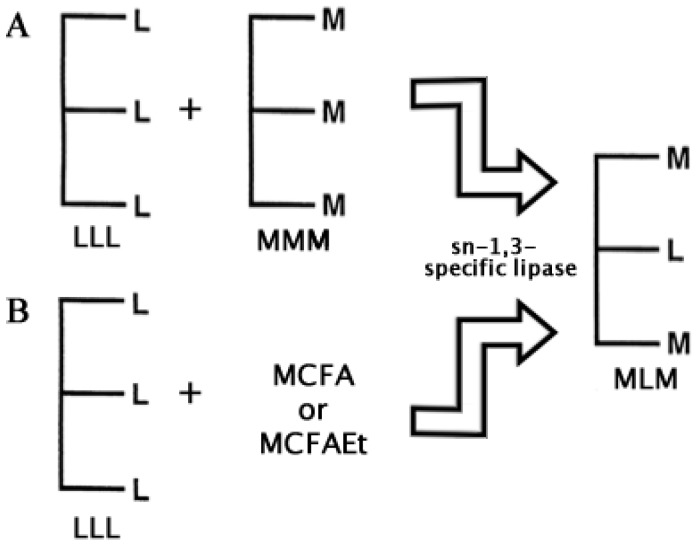
The main two approaches to synthesize MLM Structured Lipids enzymatically: (**A**) Transesterification and (**B**) Acidolysis. Subproducts like LML Structured lipids may also be obtained, but only the product of interest is shown. L = Long Chain; M = Medium Chain; MCFA = Medium Chain Fatty Acid; MCFAEt = Medium Chain Fatty Acid Ester (see also [48]).

Two basic approaches have been followed to carry out the enzymatic synthesis of MLM SLs: (1) Transesterification and (2) Acidolysis [48]. Lipase transesterifications for the synthesis of MLM SLs have been performed using different vegetal and fish oils as source of essential long PUFAs, and coconut and palm kernel oil as a source of medium chain fatty acids (C6:0–C12:0) to be incorporated in the *sn*-1 and *sn*-3 positions [42,49]. Thus, to synthesize MLM SLs, *sn*-1 and *sn*-3, regiospecific lipases are favored. In Figure 4 these two approaches are depicted using a *sn*-1,3-specific lipase.

Casas-Godoy *et al*. [50] incorporated caprilyc and capric acid into olive oil using *Yarrowia lipolytica* immobilized on Accurel. Their optimized reaction allowed them to obtain an incorporation rate of 25% and 21% for capylic and capric acid respectively within 2 days at 40 °C and 5% (w/w) enzyme load.

In another work, castor oil was dehydrated, isomerized and then transesterified with methyl laurate using *Thermomyces lanuginosa* and *Carica papaya*. The incorporation and transesterification yields were 59% and 88% for *T. lanuginosa* and 44% and 67% for *C. papaya*, respectively. This was achieved in 72 h at 60 °C with a 10% (w/w) enzyme load. Transesterification in the *sn*-2 position was observed and accounted for less than 8% [51].

Palm oil trolein has been subject to enrichment in its *sn*-2 position with DHA and ARA through acidolysis using Novozym 435. The research Group achieved an incorporation of 11% of DHA at *sn*-2 position (17% total) and 5.5% of ARA at *sn*-2 position (7% total) in 24 h at 60 °C and 10% enzyme load [52]. In a similar work, Khodadadi *et al.* [49] transesterified palm oil with tricaprylin in order to modify the former. Their results showed that Linolenic acid chains in palm oil are more easily transesterified than Linoleic or Oleic. Their best results were around 20% incorporation of caprylic chains into *sn*-1, *sn*-2 and *sn*-3 positions of which around a half corresponded to *sn*-2 position. The experiments were carried out with Novozym 435, Lipozyme TL-IM, Lipozyme RM-IM and Amano DF, though the best results were with Novozym 435, 24 h of reaction time, 50 °C and 1% enzyme load. Tecelão *et al*. [53] recently achieved 21% and 8% of incorporation of oleic acid and DHA respectively, to tripalmitin using *Carica papaya* latex at 60 °C, in a solvent-free system during 24 h.

Nagao *et al.* [54] achieved almost 100% of fatty acid incorporation with a DHA-rich by-product waste from a tuna oil industrial, hydrolysis process employed to extract DHA. Their reaction conditions were 30 °C, 120 h, vacuum at 2 kPa and 10% of Novozym 435 load. The degree of esterification with DHA was 51% and 17% at *sn*-1,3 and *sn*-2 position respectively. 

## 3. Enzymes in the Bulk Chemistry Industry

Although industrial biotransformations are mainly used for the production of fine chemicals, there are a few examples where Biocatalysis is also used to produce commodity chemicals such as acrylamide, and biodiesel. The use of biocatalysts employed to assist in synthetic routes to complex molecules of industrial interest is growing steadily [55,56,57].

### 3.1. Biodiesel

The lipase-catalyzed synthesis of biodiesel offers advantages over conventional methods for its production: lipases are able to work in gentle conditions and with a variety of triglyceride substrates, including waste oils and fats, corrosion problems present in chemical synthesis are avoided in the lipase-catalyzed synthesis, easy recovery of biocatalyst and glycerol, high levels of Free Fatty Acids (FFA) may also be esterified by lipases, and low environmental impact [58,59]. Furthermore, the separation and purification step of biodiesel is easier [60,61], resulting in a more attractive technical and environmental process. Numerous lipases have been studied for biodiesel production, with diverse triglyceride substrates and acyl acceptors. The use of edible oils is today not an option due to the food *vs*. fuel issue, which also makes them a more expensive raw material. Research studies so far have converted a series of non-edible fats and oils replacing the traditional alkaline transesterification. Process flow, reactor design and cost performance have been some of the main elements studied. It has been shown that high productivity, enzyme reuse and low reaction times can be achieved [62,63,64]. Nevertheless, enzymatic biodiesel still faces technical and economic challenges and has room for improvement to make enzymatic biodiesel a more attractive option for industrial production.

**Figure 5:**
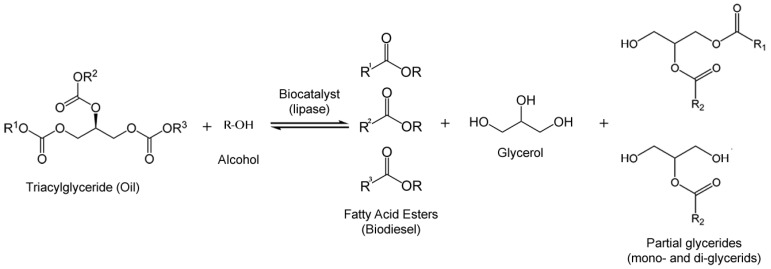
Enzymatic reaction of transesterification of oils with alcohol to produce fatty acid esters (biodiesel).

The most used method to produce enzymatic biodiesel is by transesterification of oil with an alcohol as acyl acceptor (Figure 5). Acyl acceptors, pose a challenge because the most available and cheap ones for industrial production and those who meet international specifications for biodiesel, are methanol and ethanol, which exert a strong denaturating action towards lipases [65]. Nevertheless, relatively successful efforts to overcome this inconvenience have emerged, like a pioneering work on stepwise alcohol addition [66], acyl acceptor alterations [67,68] and solvent engineering [69,70] which have sorted out this problem with relatively efficacy. Isopropanol [71], Isobutanol [72], 1-butanol [71], 2-butanol [72], 2-ethyl-1-hexanol [73], methyl acetate [74] and ethyl acetate [68] have been reported to be used as acyl acceptors. It was later documented that glycerol, a by-product of biodiesel synthesis, exerted lipase inhibition as well, which is more likely due to mass transfer limitation in the immobilized lipase [75].

Free and immobilized lipases of fungal and yeast origin such as *Mucor* sp., *Rhizopus* sp., *Candida* sp., *Aspergillus* sp., *Thermomyces* sp., *Penicillinum* sp. *and Pseudomonas* sp. have shown to be good biocatalysts for biodiesel synthesis as yields for most of them are reported to be 80%–95% [58]. Enzyme immobilization is a cost which greatly influences the economic viability when scaling-up industrial biodiesel projects; thus, alternative methods of preparing the enzyme is a current active topic in the field [76].

Organic solvents have been used to solve problems like substrate solubility and mass transfer [63]. Hydrophobic organic solvents have been used because they improve trygliceride and fatty substrates solubility [77], but leave viscous glycerol insoluble, which poses mass transfer limitations as stated above. Despite the denaturing effects of hydrophilic organic solvents over enzymes [78,79], some solvents like 1,4-dioxane and *tert*-butanol have been proven to generate high transesterification yields [69,70,80]. Incubation of lipases in alcohols with carbon number ≥3 has been documented to improve enzymatic activity in the synthesis of methyl esters [81]. Acetone has also been tested; however, the maximum yields of biodiesel have reached 40% [82]. 

The use of waste animal fat from slaughterhouses as raw material for the production of biodiesel by transesterification with ethanol was recently evaluated by Rivera *et al*. [69]. Enzymes N435 and Lipozyme RM IM at 45 °C at a 2% (w/w) enzyme load, in an organic (*tert*-butanol) and solvent-free systems were used. The biodiesel yields were 80% for the solvent-free system after 2 days of reaction, and 65% for the organic system after 1 day of reaction.

Recently, solvents like supercritical carbon dioxide have been used and biodiesel yields accounted for as much as 81% at 60 °C with a 15% (w/w) enzyme load during 4 h [83]. Ionic liquids have also been used for biodiesel synthesis [84,85] and one of the best results was achieved after 12 h at 50 °C in 1-ethyl-3-methylimidazolium trifluoromethane-sulfonate ([C2mim][OTf]) with an 80 % yield [86]. 

Intracellular lipases in the form of whole-cell biocatalysts, present a feasible alternative for biodiesel production as well. Their main advantage is the lack of laborious and costly extraction and purification procedures needed when an isolated, free or immobilized, enzyme is used [87]. In order to use whole-cell Biocatalysis, immobilization is advocated. One of the most accepted whole-cell biocatalyst systems for industrial applications is the use of filamentous fungi immobilized onto biomass support particles (BSPs) [88,89,90]. A*spergillus* sp. and *Rhizopus* sp., have been identified as robust for whole-cell immobilization, and thus have become the most widely studied fungi for biodiesel production [58]. Oils from yeasts and fungi are an alternative source of oils for biodiesel, as they can accumulate long-chain triacylglycerols with 16 and 18 carbons. The oil contents obtained from several yeast strains such as *Cryptococcus*, *Lipomyces*, *and Rhodotorula* species have reached 60%–70% of their dry weight [91].

### 3.2. Industrial Polymers

Biocatalysis has opened new synthetic strategies for organic chemists because it has been applied to synthetic chemistry in order perform stereochemical complex reactions in a relatively simple, straightforward way, which otherwise would require laborious protection-deprotection steps, toxic reactants and/or high-temperature/pressure procedures [92,93]. Thus, these advantages of using enzymes have been adopted in the field of polymer synthesis, in which most polymers are difficult to produce or to control by conventional chemical methods. Polymers with specific structures can be prepared enzymatically. In contrast, attempts to attain similar levels of polymer structural control by conventional chemical methods may prove to be a true challenge. From an environmental stand point, polymers derived from enzyme-mediated catalysis, whether polyesters, polyphenols, polysaccharides, proteins, or other polymers, are in most cases biodegradable, a feature highly sought after these days.

The most common type of enzymes which are capable to catalyze polymerization reactions are: Transferases, Oxidoreductases and Hydrolases. 

#### 3.2.1. Transferases

Transferases transfer a glycosyl group from a sugar nucleotide donor to specific nucleophilic acceptors. Despite they impose a high degree of regio- and stereochemical control to the glycosidic bond they form, and thus having a great synthetic potential for interesting polymeric materials, their use for synthesis *in vitro* is limited and even prohibitive by both, the availability of enzymes due to expression and solubility, and by the high costs of expensive activated donor sugars such as uridine diphosphate. They are very sensitive biocatalysts so their isolation and use on a larger scale is not practical, and they are mostly reserved for specialty research endeavors [94].

#### 3.2.2. Oxidoreductases

On the other hand, Oxidoreductases and particularly Hydrolases are more robust enzymes, less sensitive and easier to obtain and use *in vitro*. Polymers typically produced by these three types of enzymes are shown in Table 1.

**Table 1:** Common enzymes and the typical polymers synthesized by them.

Enzymes	Polymers synthesized
Oxidoreductases	
*Peroxidases* *Laccases* *Tyrosinases* *Glucose oxidases*	Polyphenols, polyanilines, vinyl polymers
Transferases	
*Glycosyl transferases* *Acyl transferases*	Polysaccharides, cyclic oligosaccharides, polyesters
Hydrolases	
*Glycosidases* *Lipases* *Peptidases* *Proteases*	Polysaccharides, polyesters, polycarbonates, polyamides, polythioesters, polyphosphates

##### 3.2.2.1. Polyaromatics

Polyaromatics are widely found in nature such as lignin, and flavonoid compounds. Much of their role in nature is as structural component conferring structural strength to living systems, in the case of lignin, and as a bioactive molecule in the case of flavonoids. Phenol is the single aromatic most important compound for industrial applications. Current polymeric materials produced commercially from phenolic compounds as industrial plastics include Bakelite and poly-2,6-dimethyl-1,4-phenylene oxide) (PPO) which bear good toughness and temperature-resistant properties. However, the oxidative polymerization of phenol with a conventional catalyst usually gives insoluble products with uncontrolled structure [95]. Moreover, as Bakelite and PPO are phenol-formaldehyde resins, the toxicity of formaldehyde has brought limitations on their industrial production. 

On the other hand, an enzyme-catalyzed polymerization, offers not only the typical benefits of Biocatalysis, but the possibility to have a better control over polymerization [96]. 

In a recent work, Salvachúa *et al.* [97] used Versatil peroxidase (VP) to crosslink several monomeric lignans and peptides. Results show lignan oligomers of a maximum DP of 9 with a Number Average Molecular Weight Mn = 3,300. As per the reactions with peptides, peptide oligomers with a maximum DP of 11 and a Mn = 6,500 were obtained. Hetero-oligomers between lignans and peptides were also synthesized by VP. Oligomers of a DP = 6 and a Mn = 2,300 were obtained where the ratio peptide: lignan was 1:5. The reactions were done at room temperature with 1.5 U mL^−1^ of VP, 0.1 mM H_2_O_2_ and 0.1 mM Mn^2+^, during 24 h. 

**Figure 6:**
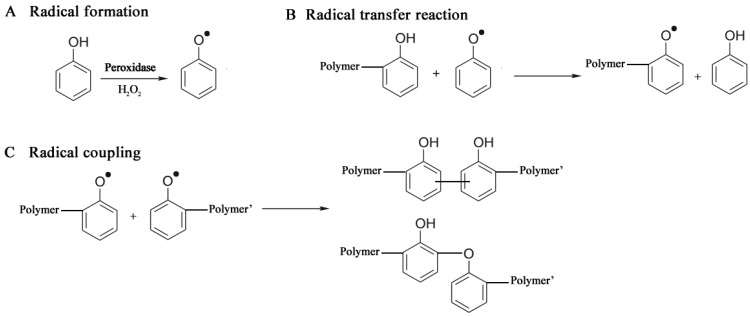
Mechanism of phenol polymer formation (see also [104]).

A peroxidase-catalyzed polymerization was performed under mild reaction conditions, using an aqueous buffer alcohol resulting in a soluble polyphenol with M_n_ of 3,000–6,000 [98,99]. The catalysts included horseradish peroxidase (HRP) and soybean peroxidase (SBP). The polymer solubility increased with increasing the oxyphenylene unit content (32%–59%) which was controlled by varying the methanol amount [100], and the solution pH [101]. Monodisperse polymer particles (250 nm diameter) were synthesized from phenol and subsituted phenols like m-cresol, p-cresol and polyphenylphenol, with HRP in a dispersion system 1,4-dioxane/buffer [102,103]. The mechanism of a peroxidase-catalyzed polymerization of phenols comprises three stages: Radical formation, Radical transfer and Radical coupling, shown in Figure 6.

It was revealed that meta-substituted phenol polymerizations catalyzed by HRP and SBP give rise both to higher yields when small and large-bulky substituents are used, respectively [105]. Cardanol, a natural phenol derived from cashew nut shell liquid (CNSL) with a C15 unsaturated alkyl meta-substituent, has good perspectives for industrial utilization such as resins, friction lining materials, and surface coatings. Therefore, an enzymatic approach to develop derivatives from this compound is attractive. Kobayashi *et al.* [106] obtained an artificial polymer structurally similar to “urushi”, a Japanese lacquer, through an SBP-polymerization of Cardanol. The polymer had a molecular weight between 2,000–4,000 Da., soluble in polar organic solvents and possessed a tough and hard property as a film with a glossy surface finish. Kim *et al*. [107] also synthesized polycardanol with SBP and found it had better anti-biofouling activity to *Pseudomonas fluorescens* compared to polypropylene.

Various other m-substituted phenols have been polymerized by HRP and SBP, for example meta-alkylphenols, meta-halogenated phenols, and meta-phenylphenol [108]. These enzymatically synthesized m-substituted polymers were applied to positive-type photoresists for printed wire boards, because of their high solubility toward alkaline solution and high thermal stability [108,109]. 

*Ortho-*substituted phenols have been polymerized with SBP affording a variety of oligophenols (dimers to pentamers) and some of their oxidation products, including quinones and demethylated quinones. Some of these are considered to serve as biologically important compounds with therapeutic potentials [110]. In separate reaction systems, it was possible to synthesize poly(phenylene oxide) (PPO) from two different *o*-substituted phenols, 2,6-dimethylphenol and 3,5-dimethoxy-4-hydroxybenzoic acid (syringic acid), using HRP, SBP and a laccase [111,112,113]. The polymers were formed exclusively of 2,6-dimethyl- or 2,6-dimethoxy-1,4-oxyphenylene units respectively, which are similar in structure to a widely used high-performance engineering plastic PPO. 

Para-alkylphenols were polymerized by HRP in an aqueous 1,4-dioxane solution to synthesize polymers with Mn of several thousands, whereby the polymer yield increased as the n-alkyl chain length increased from 1 to 5 [102,114]. Oxidative polymerization of natural *para*-substituted phenols like tyrosine ethyl ester or methyl ester or phenol derivative hydroquinone-β-d-glucopyranoside (arbutin) [115] were carried out using HRP catalyst in an aqueous buffer solution. A mixture of phenylene and oxyphenylene units was observed in the tyrosine polymer, whereas the arbutin polymer, after degycosilation, consisted of C-C *o*-position hydroquinone units. Resulting polymers had a molecular weight from 1,500–4,000. Reactions catalyzed by HRP proceeded, involving radical species, yet took place chemoselectively. *Para*-phenylphenol is one well-studied *para*-substituted phenols in peroxidase-catalyzed polymerizations. Polymers up to 26,000 Da have been synthesized with HRP [99]. However, the polymerization of *para*-phenylphenol lacks precise structure control due to the existence of both stabilized *ortho*- and *para*-single electron radical species, causing different kinds of linkages and leading to a complex structural polymer [116]. 

Other numerous reports manage different aspects of the enzymatic polymerization of phenols: use of mixtures of aqueous-organic solvents with 1,4-dioxane, methanol, DMF [117], or total organic systems (isooctane) [118], ionic liquids [119], using cyclodextrins as a phenol solubilizing agent [120], incorporating PEG as a template aid [121], or carrying out the reaction in micelles and reverse micelles [122] or in capsules [123].

An important group of natural phenols widely distributed throughout the plant kingdom are flavonoids. Flavonoids are benzo-γ-pyrone compounds consisting of phenolic and pyrane rings. Their biological and pharmacological effects as antioxidant, antimutagenic, anticarcinogenic, antiviral and antiinflammatory have been extensively reviewed [124,125,126,127].

Flavanols or commonly known as catechins are the major group of polyphenols present in green tea. The main catechins in green tea are (+)-catechin, (−)-epicatechin (EC), (−)-epigallocatechin (EGC), (−)-epicatechin gallate (ECG), and (−)-epigal-locatechin gallate (EGCG). Poly(cathechin) has been synthesized by HRP in aqueous-organic solvents having yields of DMF-soluble polymers around 30% and molecular weights of 3,000 [128]. It has also been polymerized by a laccase derived from *Myceliophthore* (ML) in a mixture of acetone-buffer pH 5 rendering an 8,000 Da polymer [129]. In this case, as in other phenol polymerizations [100], the organic solvent/buffer ratio greatly affected the solubility of the synthesized polymer. The antioxidant properties of catechins and ECGC, not only remained, but increased in a concentration dependent manner in poly(catechin) and poly(ECGC) synthesized by HRP and ML [129,130]. Superoxide anion scavenging activity of poly(ECGC) was much superior than monomeric ECGC and poly(catechin) [130]. Inhibitory activity of Xanthine oxidase, which was negligible in monomeric catechin or ECGC, was markedly observed in poly(catechin) and in poly(ECGC), inclusive was higher than allopurinol, a commercial inhibitor frequently used for gout treatment [129,131]. Recently, a laccase was used to polymerize catechin in order to attach it to biomedical catheters in order to reduce bacterial biofilm formation [132].

Rutin has also been polymerized in organic-aqueous systems by Laccase ML-mediated catalysis. Yields superior to 70% were mostly observed and molecular weights between 7–9 kDa were registered [133]. 

Flavonols (quercetin) and isoflavones were also subject to an enzymatic oxidative polymerization by HRP and SBP in aqueous-organic solvent systems. Weight Average Molecular Weight Mw and Number Average Molecular Weight Mn for poly(quercetin) was 10,000 and 2,500 respectively, with a yield around 50%. Poly(rutin), poly(catechin), poly(daidzein), poly(formononetin) and others were also obtained in similar numbers [128].

Functionalization of polymers with flavonoids has also been investigated. Quercetin-functionalized chitosan has been the most studied system to yield antimicrobial products [134,135,136,137]. However, naringin-PVDF, morin-resin, catechin-inulin systems have also been reported [138,139,140].

#### 3.2.3. Hydrolases

Hydrolases are the enzyme class with most widespread use and applications in the world. Around 75% of all commercialized industrial enzymes are hydrolytic in action. Despite proteases and carbohydrases are the most demanded subclass of enzymes accounting for around 40% of world’s enzyme sales, lipases remain as the biocatalyst subclass most widely used in organic chemistry [141,142].

Lipases have been used to catalyze reactions in polycondensations of dicarboxylic acids with diols [143], ring-opening polymerization (ROP) of lactones [144], cyclic carbonates [145] and polycondensations of hydroxycarboxylic acids [146]. More examples are described in the following sections.

#### 3.2.4. Polyesters

Polyesters occupy the 4th place among the most important biomacromolecules in living systems. They are macromolecules with good biodegradability, biocompatibility and permeability, and thus are highly suitable for biomedical applications, such as implant biosorbable materials, for tissue engineering and delivery vehicles for drug or genes [147]. Polyesters are very valuable industrial materials that are broadly used like poly(ethylene terephthalate) (PET), poly(butylene succinate) (PBS), poly(ε-caprolactone) (PCL), and poly(lactic acid) (PLA). 

Two major approaches can be taken to perform enzymatic polyester synthesis: (1) polycondensation and (2) ring-opening polymerization (ROP). PET and PBS are produced in large-scale by polycondensation whereas PCL and PLA are produced via ROP. Classical cationic, anionic or metallic chemical synthesis of polyesters may render them unsuitable for biomedical applications due to the toxicity of these reagents. Enzyme catalyzed synthesis of esters has circumvented this and other problems since enzymatic reactions do not require high energy input (temperature or pressure), most enzymes can carry out regio-, chemo- and enantiospecific reactions with no need of protecting groups, exhibit high catalytic activity, they may be reused and as a consequence of all this, reactions are environmentally and health safer than the conventional chemical route [148].

#### 3.2.5. Polycondensations

##### 3.2.5.1. Lipase-Catalyzed Polycondensations

In lipase-catalyzed polycondensations dicarboxylic acids are esterified with diols to produce polyesters. Reactions have been realized mostly in organic solvents, in solvent-free media and in aqueous systems. However, there have been studies in ionic liquids [149] and supercritical fluids as well [150]. In the early years of exploration of these systems, Okumura *et al.* [151] set a series of reactions with dicarboxylic acids and diols in aqueous systems using a lipase from *Aspergillus niger* and he obtained oligomers of 3–7 units. Extracting water molecules from the system, either with molecular sieves or applying vacuum, seemed to improve the polymer size up to a DP of 20 in an adipic acid-1,4-butanediol reaction [152], and up to a Mn = 77 kDa in an adipic acid-1,6-hexanediol reaction [153]. Organic solvents with high boiling points were identified to favor the polyester synthesis. In an aim to study natural hydroxy-acids, Gómez-Patiño *et al.* [154] obtained oligomers from tomato cuticle monomer, 10,16-dihydroxyhexadecanoic acid and its methyl ester. *Candida antarctica* B was the best performer among the 5 lipases tested in organic media reactions obtaining the largest oligomer with a DP = 3 and Mw = 1,206.

Reactions between dicarboxylic acids and diols conducted in water with *Candida antarctica* (N435), but with a continuous dehydrating process, are claimed to give rise to good yields [155].

Polycondensations with structurally different monomers have been realized, for example siloxane-containing diacids with diols (Mw = 20,000) [156], diacids with glycerol (Mw = 2,000–6,000) [157,158], thio-glycerol with diacids (Mw = 170,000) [159], diacids with a variety of sugars, sorbitol, erythritol, xylitol, ribitol, mannitol, glucitol, galactitol yielded polyesters with Mw = 10 kDa for galactitol and 75 kDa for Mannitol. The regioselecitivy of the sugar esterifications was mostly consistent in all the sugars to be on the primary hydroxyl groups [160].

Activation of dicarboxylic acids has been employed by means of esterification the carboxylic groups with methanol or ethanol [161], by esterification [162] or by vinyl activation [163]. Azim *et al*. [164], in an interesting strategy, firstly polymerized succinic acid with 1,4-butanediol to obtain a cyclic oligomer, which later was repolymerized by ring-opening to obtain a high molecular weight polymer (Mw = 130 kDa). Activation of dicarboxylic acids by means of esterification the carboxylic groups with halogenated alcohols like 2-chloroethanol, 2,2,2-trifluroethanol and 2,2,2-trichloroethanol lead to an increase of Mw by a factor of 5 [162]. However, the halogenated alcohols produced as a consequence of the reaction may damage the biocatalyst. Activation of carboxylic groups by vinyl esterification produced in most cases polyesters with Mw around 25 kDa [103,165,166].

Through reaction optimizations, Yao *et al*. [167] were able to achieve a polyester Mw = 16.6 kDa using 1,8-octanediol, adipic acid and l-malic acid as monomers. The incorporation of a sugar to be used as a third monomer is a likely means to modify the properties of conventional polymers, and an interesting strategy to prepare a variety of functional materials. 

Recently it was reported the polymerization of β-alanine by a lipase catalyzed polycondensation reaction between β-alanine esters. Lipase B from *Candida antarctica* aggregated in CLEAs was the biocatalyst that converted β-alanine esters into polymers in high yield (up to 90%) and with high DP (up to 53 units). The best results were obtained by using methyl esters and methyl-tert-butyl ether (MTBE) as the solvent medium at 50 °C for 16 h [168].

##### 3.2.5.2. Protease-Catalyzed Polycondensations

Proteases, like some other hydrolases, catalyze not only hydrolytic reactions (peptide hydrolysis in this case) but also peptide bond formation under appropriate conditions to give polypeptides. There exist several oligopeptides with varied biologically active properties that have been synthesized widely using proteases as catalysts via condensation reactions [169]. Some examples include, aspartame synthesized by thermolysin [170], oxytocin by papain, thermolysin, and chymotrypsin, and somatostatin by thermolysin, and chymotrypsin [171]. Various attempts to synthesize long-chain polyaminoamides were done by a protease-catalyzed polycondensation using chymotrypsin, trypsin, subtilisin and papain. However, only lower molecular weight polymers were obtained [172].

#### 3.2.6. Polyamides

It was recently reported the synthesis of polyamides by lipase Novozym 435 in high yields (93%) and a short lapse of time (30 min). Authors attribute this high productivity to the use of “designed” monomers of the type: ω-amino-α-alkoxy acetic acid ethyl ester, which bears an ω-amino group and an oxygen atom in β position. The reactions were performed in bulk at 80 °C with an enzyme load of 45% (v/v) and the resultant polyamides had a DP = 15 (PDI = 1.6) and a Mw between 3,000–4,000 [173].

#### 3.2.6. Vinyl Polymers

One field that has expanded tremendously in the past two decades is the enzyme-initiated radical polymerization of aromatic and vinyl monomers [149]. Vinyl monomers studied for enzymatic polymerization are basically classified into (meth)acrylic type and styryl type, which both involve the formation of a radical species. HRP and Laccase peroxidases have been reported to perform well in the meth(acrylic)-type polymerization of acrylamide. In a HRP-mediated free radical polymerization of vinyl monomers, the HRP-mediator system has three components: enzyme, oxidant (H_2_O_2_) and an initiatior, such as β-diketones which are commonly used. On the other hand, when several oxidoreductases, such as laccase, lipoxidase, and sarcosine oxidase, were used as catalysts, the acrylamide polymerization proceeded even without H_2_O_2_ or β-diketones. This was the case for laccase from *Pycnoporus coccineus* which induced the acrylamide polymerization in water at 50–80 °C under argon to produce poly(acrylamide) with Mn = 1 × 10^6^ , Mw/Mn ≈ 2 for 4–24 h and up to 81% yields [174]. Since the laccase-mediator system (LMS) may induce the radical polymerization of vinyl monomer with no H_2_O_2 _ and is environmentally benign, different conditions for polymerization with commercial laccase *Myceliophthora thermophila* were further examined and optimal conditions were found as slightly acidic reaction media at around 50 °C using β-diketones and O_2_ as the initiator and oxidant respectively. The molecular weight of poly(acrylamide) was Mn 6–28 × 10^4^; Mw/Mn = 2.5–3.2 and could be controlled through the ratio of monomer to enzyme [175].

Poly(acrylamide) polymers of Mn of 1.5–4.6 × 10^5^ with Mw/Mn 2.0–2.4, and acrylamide conversion between 70%–90% were synthesized by HRP in water at room temperature with 2,4-pentanedione as an initiator intermediate [176]. 

Enzyme-catalyzed vinyl polymerizations have been demonstrated in recent years with significant control of polymer molecular weight and yield depending on reaction conditions [149]. Resins of poly(sodium acrylate) are used as water-absorbent materials for cleaning surfaces, in water and oil conditioning, personal care products, and disposable materials for medical applications [177].

#### 3.2.7. Nylons

Polyamides, also called nylons, display improved physical properties compared with polyolefins and polyesters due to the directionally specific inter-chain hydrogen bonds and significantly enhanced melting points [178]. Nylons can be synthesized either by polycondensation or by ROP. Ragupathy *et al.* [179] presented two and three step polycondensation and ROP methods to enzymatically produce nylon-8,10, nylon-8,13, nylon-6,13, and nylon-12,13. He used 1,8-diaminooctane (DAO) and diethyl sebacate (DES) for the polycondensations and DAO and lactone ethyleneridecanoate (ETD) for ROP. Novozym 435 in diphenyl ether at 150 °C carried out the polymerizations. Monomer conversion reached 97% and 90% and polyamides synthesized had an Mw of 5,000 and 8,000 for polycondensation and ROP reactions respectively.

#### 3.2.8. Acrylate and Styrene Polymerization

Methyl-methacrylate (MMA) was polymerized by en enzyme-catalyzed reaction using the ternary system (enzyme, oxidant, and initiator) in water and water-miscible organic cosolvents such as DMF, acetone, dioxane, and THF. Soybean peroxidase in aqueous solution afforded a 48% yield of poly(methyl methacrylate) (PMMA) with Mn = 9.3 × 10^5^ and Mw/Mn = 6.8. When HRP II (type II) was used, a 45% yield of PMMA was obtained with Mn = 6.3 × 10^5^ and Mw/Mn = 3.0. It was found that yields of PMMA increased when cosolvents with low dielectric constant like dioxane and THF were used [180]. 

Respect to styrenic polymerizations, HRP in water was used to polymerize styrene and some derivatives. The polymerization was investigated using several β-diketones as initiators, and proceeded in a mixed solution of H_2_O/THF = 3 (v/v). Polystyrene synthesized using 2,5-cyclopentanedione as initiator resulted in 60% yield with Mn and Mw/Mn of 6.7 × 10^4^ and 1.9 respectively. Polymer yield, Mn and Mw/Mn of the resulting polystyrene depended on the type of initiator used. Synthesis of polymers from styrene derivatives, 4-methylstyrene and 2-vinylnaphthalene were also investigated and afforded polystyrenes in a >90% yield of polymer with a Mn of 1.15 × 10^5^ and Mw/Mn = 2.28 [181].

#### 3.2.9. Ring-Opening Polymerizations (ROP)

Among the two major synthetic polymerization approaches, ring-opening polymerization (ROP) has been most extensively studied to polyester synthesis.

Polymerization of lactones via ring-opening catalysis carried out by lipases has been studied since the early 1990s. This catalytic activity for ROP of lactone monomers has been the research field on diverse lipases such as *Pseudomonas fluorescens*, *Pseudomonas cepacia*, *Candida rugosa*, *Candida cylindracea*, *Candida antarctica*, *Aspergillus niger*, *Mucor miehei*, *Penicillium roqueforti*, *Rhizopus japanicus. Yarrowia lipolytica*, *Carica papaya* and porcine pancreas lipase [182,183,184,185,186,187,188,189]. Cutinase from *Humicola insolens* (HiC) has also been reported to carry out ROP [190]. 

Poly(1,4-dioxan-2-one) (polyDO) is a desirable biocompatible polymer with good flexibility and tensile strength which might be considered for biomedical applications. This polyester was synthesized by ROP of 1,4-dioxan-2-one by lipase B from *Candida antarctica* at 60 °C, resulting in a polymer of Mw = 41,000 [191]. In another study, Jiang *et al*. [192] claimed to synthesize by ring-opening copolymerization a polyester from 1,4-dioxan-2-one (DO) with pentadecalactone (PDL) to give a copolyester of poly(DO-co-PDL) with Mw > 30,000. They used *Candida antarctica* B lipase in toluene or diphenyl ether at 70 °C for 26 h. 

Lipase 2 from *Yarrowia lipolytica* and *Carica papaya* latex were recently employed to polymerize ε-CL in bulk. Lipase 2 from *Y. lipolytica* was found to be very efficient as catalyst for several reactions [193] and latex from *C. papaya* is a low-cost auto-immobilized biocatalyst. The polymerization yields were 74% and 40% with an Mw of 1,350 and 1,100 respectively. Reactions were realized at 150 °C and 6 h for *Y. lipolytica* and 70 °C and 24 h for *C. papaya* [182].

As to macrolides, lipase *Pseudomonas fluorescens* was able to polymerize 11-undecanolide (12-membered, UDL), 12-dodecanolide (13-membered, DDL), 15-pentadecanolide (16-membered, PDL), and 16-hexadecanolide (17-membered, HDL) [114,186,187,188,189,194,195,196]. Lipases from *Candida cylindracea* and *Pseudomonas fluorescens* catalyzed the ROP of UDL in bulk with quantitative yields affording polyesters of Mn of 23,000 and 25,000 [197,198]. In another study, ROP by lipases CA, lipase CC, lipase PC, lipase PF, or PPL in bulk at 45–75 °C for 5 days was done over HDL, the largest unsubstituted lactone monomer studied so far. The study showed the synthesis of polyHDL with Mn reaching to 5,800 in high yields [199]. It was discovered that a larger ring monomer, like PDL showed a greater reactivity towards polymerization than a smaller monomers like 1,4-polydioxan-2-one (DO) [192].

Ebata *et al*. obtained a poly(ε-CL) from a cyclic dimer of ε-CL (14-memberd) that was polymerized at 70 °C in toluene by lipase B from *Candida antarctica*, affording quantitatively a poly(ε-CL) with Mn of 18,000 [200]. This research Group, under the same strategy used to produce oligomers which are then subject to subsequent polymerization, claimed that a poly(butylene-succinate) (PBS) polyester of Mw 130,000 was obtained from cyclic dimers of butylene-succinate of Mn = 390. The reaction was carried out in toluene at 120 °C using 40% (w/w) of lipase *Candida antarctica* B for 24 h [201].

It was found that among ε-CL substituted monomers, ω-methyl ε-CL showed the least reactivity towards the lipase-catalytic ROP [202]. On the other hand, α-methyl or δ-methyl ε-CL afforded polymers of Mw = 8,400–11,000 with yields of 74% and 93% respectively [203].

An equilibrium phenomenon was observed between the synthesis of cyclic and linear polymers in a lipase-catalyzed ROP reaction of lactones. Cyclic oligomers in addition to major linear polyesters coexist in equilibrium [204,205]. This cyclic-linear equilibrium is solvent-dependent, for example Novozym 435 was used to catalyze the ROP of ε-CL at 60 °C in bulk or in an organic solvent. In bulk, the polymer products coexisted as a cyclic dimer of ε-CL and linear poly(ε-CL) in 2% and in 98% abundance, respectively. When the reaction was carried out in in acetonitrile, THF or 1,4-dioxane the same proportion was observed, as for example the equilibrium yields in acetonitrile were 70% for the cyclic oligomers (17% of dilactone and 53% of cyclic oligomers) and 30% for linear poly(ε-CL). When the reaction was carried out in isooctane, the proportion inverted to 3% and 97%, respectively, as in the bulk reaction [204]. 

Another hydrolase enzyme, a depolymerase, was found to catalyze ROP reactions *in vitro*. This depolymerase is a poly(hydroxybutyrate) (PHB) (EC 3.1.1.75), and was extracted from *Pseudomonas stutzeri*, *Alcaligenes faecalis* and *Pseudomonas lemoignei*. *A. faecalis* PHB depolymerase was assayed with a series of small, medium and large lactones and the best polimerization activity (93% yield) was obtained with β-butyrolactone, resulting in the formation of polyesters with a Mw = 16,000. On the other hand, medium and large lactones (ε-caprolactone, 11-undecanolide, and 12-dodecanolide), which are readily polymerized by lipases, were scarcely polymerized by PHB depolymerase [206].

### 3.3. Commodity Chemicals

**Figure 7:**
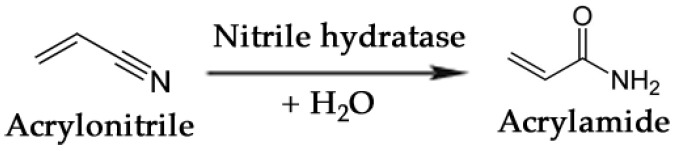
Enzymatic conversion of acrylonitrile to acrylamide by means of a nitrile hydratase.

One of the most noteworthy cases of biocatalytic production of a commodity chemical is the bioconversion of acrylonitrile to acrylamide [207]. This reaction, schematically simplified in Figure 7, is carried out by a nitrile hydratase whose catalytic activity transforms a cyanide group into an amide. Mitsubishi Rayon Co., Ltd. (Tokyo, Japan) currently produces over 20,000 tons annually of acrylamide using a third-generation biocatalyst, *Rhodococcus rhodochrous* J1, developed for commercial use by Nitto Chemical Industries. Acrylamide is produced from acrylonitrile in a continuous system of fixed-bed reactors at 10 °C with polyacrylamide-immobilized J1 cells. The process achieves conversion of acrylonitrile to acrylamide in ~99.9% yield, and the catalyst productivity is >7,000 g of acrylamide per g dry cell weight [208].

**Figure 8:**
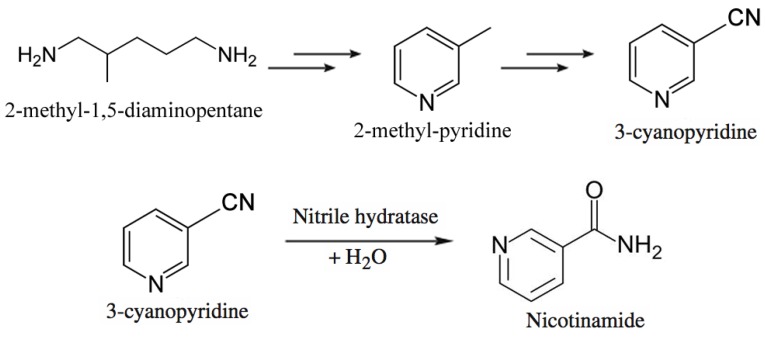
Chemoenzymatic route to obtain nicotinamide developed by Lonza [209,210].

Lonza Guangzhou Fine Chemicals produces nicotinamide through a chemoenzymatic process shown in Figure 8 [209,210]. They start from 2-methyl-1,5-diaminopentane, a byproduct from production of nylon-6,6, which is converted to 3-methylpyridine, which in turn is ammoxidized. The resulting 3-cyanopyridine is hydrolyzed to nicotinamide using immobilized *Rhodococcus rhodochrous* J1 cells [211,212]. The Plant’s capacity is 3,400 tons per year of nicotinamide. The enzymatic process affords the desired amide at >99.3% selectivity at 100% conversion, whereas the chemical process requires caustic hydrolysis of 3-cyanopyridine but also produces 4% nicotinic acid as byproduct. 

A commodity chemical which is aimed to substitute to some extent the universally oil-derived terephthalic acid is 2,5-furandicarboxylic acid (FDC) [213]. Terephthalic acid is one of the main raw materials to produce polyesters. FDC relies in the supply of 5-hydroxymethylfurfural (HMF), which is the product of the dehydration of hexoses. Oxidation of HMF yields FDC and may be realized by using heterogeneous or electrochemical catalysis [214,215]. However, two biocatalytic processes have been devised to help in having a reliable and sustainable supply for HMF: a whole-cell biocatalytic approach using a recombinant *Pseudomonas putida* hosting an oxidoreductase from *Cupriavidus basilensis.* This process is able to produce 30 g/L of 2,5-furandicarboxylic acid from HMF having a yield of 0.97 mol/mol [216]. The productivity of the whole process is 0.21 g/(L∙h) under aerobic fed-batch conditions. The second approach consists of the use of a chloroperoxidase and *C. antarctica* lipase B [217,218].

## 4. Enzymes in the Fine Chemicals Industry

The role of Biocatalysis in the pharmaceutical and fine-chemical industries is clearly expanding. There are estimated to be around 150 implemented biocatalytic processes in industry, and the majority of these are in the pharmaceutical sector [219]. As stereoisomerism is quite relevant in pharmaceutics and fine chemistry, and a single swap in hydrogen position may mean a great difference in the bioactive function of a compound, Biocatalysis fits well to meet these challenges. Besides the enantio-, regio- and chemoselectivity that enzymes exhibit, their ability to perform complex catalysis procedures in a simple step, which otherwise, under a chemical approach, might require laborious treatments, toxic reagents or high energy input, has provided a strong basis for their usage in the fine chemicals industry. Some examples of the contribution of Biocatalysis to this field are described below.

A precursor of aspartame, *N*-(benzyloxycarbonyl)-l-aspartyl-l,-phenylalanine methyl ester (Z-APM) has been synthesized from *N*-(benzyloxycarbonyl)-l-aspartic acid (Z-l-ASP) and l-phenylalanine methyl ester (l-PM) with thermolysin [220]. By reverting the peptide-bond hydrolytic nature of thermolysin, this precursor is synthesized on a multi-thousand ton per year scale by a DSM/Tosoh joint venture (Holland Sweetener Company, Geleen, The Netherlands).

Pyrethroids now constitute the majority of commercial household insecticides. A novel industrial application of a lyase enzyme is to produce a pyrethroid intermedieate, (*S*)-*m*-phenoxy-benzaldehyde cyanohydrin (sPBC). Hydroxynitrile lyase, is an enzyme that catalyzes the addition of HCN to aldehydes and ketones [221]. Thus, the production of sPBC from *m*-phenoxybenzaldehyde by hydroxynitril lyase has been carried out on a multi-ton scale by DSM.

l-carnitine is a quaternary ammonium compound biosynthesized from the amino acids lysine and methionine [222]. Within mammal cells, l-carnitine is required for the transport of fatty acids from the cytosol into the mitochondria during the lipids oxidation [223]. Due to this role in lipid oxidation, l-carnitine is often advertised to improve fat metabolism, reduce fat mass, and increase muscle mass, which is widely available as an over-the-counter nutritional. l-carnitine is produced by Lonza (Basel, Switzerland) on an industrial scale using an enzyme from strain *Agrobacterium* HK1349 in a whole cell biotransformation process [224]. The process actually comprises a dehydrogenation of γ-butyrobetaine to 4-(trimethylamino)-butenoic acid followed by selective addition of a water molecule by l-carnitine lyase.

l-*tert*-leucine is an important chiral building block and intermediate of many ligands in chemo-catalysis in the pharmaceutical industry [225]. Menzel *et al*. [226] presented a process to produce l-*tert*-leucine in a whole-cell Biocatalysis system using a recombinant *E. coli* host expressing the two enzymes required by the process: a leucine hydrogenase and a formate dehydrogenase. The former catalyzes the main reaction, reductive amination of an α-keto acid, and the latter serves as a NADH regenerator. A yield of 84% in 24 h and an enantiomeric purity of ee > 99% was obtained. The process has been scaled up to tons level. Degussa-Hüls has used a similar whole-cell Biocatalysis system to produce l-*tert*-leucine, which has taken to a commercial scale [227].

Lonza produces 5-methylpyrazine-2-carboxylic acid on a commercial scale from the *p*-xylene analogue 2,5-dimethylpyrazine. This compound is used as a blood-lowering drug of the sulfenyl urea class commercialized as Glycotrol. The synthesis is carried out in *P. putida* ATCC33015 as a whole-cell biocatalyst, expressing a series of enzymes (a monooxygenase and two dehydrogenases) [224].

Japan Energy (Saitama, Japan) produces alkyloxiranes and phenyloxiranes on an industrial scale, especially the chiral building block 2-(trifluoromethyl)oxirane from 3,3,3- trifluoropropene through an oxidative reaction, using a whole-cell biocatalyst from *Nocardia corallina* [228]. 

Vanillin, the main component of vanillin extract, a natural flavoring, whose demand has long exceeded the supply, should be synthesized chemically from guaiacol, in order meet demand. Nevertheless, a whole-cell biocatalytic synthesis of vanillin from glucose has now been elaborated. Glucose is transformed into vanillic acid by a recombinant *E. coli* biocatalyst under fed-batch fermentor conditions. Then reduction of vanillic acid to vanillin is carried out by aryl aldehyde dehydrogenase isolated from *Neurospora crassa.* The biocatalytic route avoids the use of carcinogenic reagents and non-renewable petroleum derivatives (guaiacol) [229].

Aresta *et al.* used a carboxylase in the synthesis of 4-hydroxy benzoic acid, which is an intermediate for the synthesis of preservatives. The carboxylase enzyme is extracted from the bacteria *Thauera aromatica*, and it acts over the phenyl-phosphate moeity carboxylating it with 100% selectivity and 90% yield at 300 °K, P_CO2_ = 0.1 MPa [230]. 

Alfuzosin, a quinazoline derivative, against Benign prostatic hyperplasia (BPH), is synthesized through intermediate compund tetrahydro-*N*-[3-(methylamino)-propyl]-2-furan-carboxamide. This intermediate was traditionally produced in a three-steps chemical route from 2-tetrahydrofuroic acid involving a series of chemicals and distillation. The enzyme-catalyzed reaction with lipase B from *Candida antacrtica*, afforded the intermediate in two steps in which the same enzyme catalyzed the two reactions: esterification and amidation. This biocatalytic procedure simplyfied and made more environmentally friendly the previous chemical synthesis [231].

**Figure 9:**
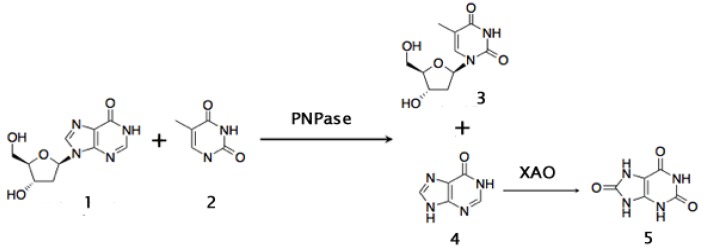
Biocatalytic synthesis of thymidine using a Purine Nucleoside Phosphorilase (PNPase) and the biocatalytical removal of hypoxantine with a Xanthine Oxidase (XAO) to obtain uric acid. Compounds: 1: 2’deoxyinosine; 2: Thymine; 3: Thymidine; 4: Hypoxanthine; 5: Uric acid (see also [233]).

Another important intermediate in the synthesis of antiviral nucleosides such as 3'-deoxy-3'-azi-dothymidine (AZT), is thymidine. Production of thymidine is currently based in the hydrolysis of DNA from natural sources. A new biocatalytic approach for its production has been developed. It consists of transfering the 2'-deoxy-ribose moiety of 2'-deoxyinosine to thymine. The purine nucleoside phosphorylase of *Bacillus stearothermophilus* was used to carry out this reaction. A xantine oxidase is added in order to convert the concomitant hypoxantine produced to uric acid, and thus draw the thermodynamic equilibrium towards the synthesis of thymidine (Figure 9). The yield of thymidine of this whole-cell biocatalytic route reached 68% under mild conditions [232].

Many catalytic functions that enzymes perform in the pharmaceutical industry is in resolution of racemic mixtures, in which one enantiomer is acylated or hydrolyzed selectively affording an easier separation of both enantiomers. One recent example of this is the resolution of pro-drug (R,S)-2-bromophenylacetic acid octyl ester. Rivera *et al.* [234] accomplished its resolution with high enantioselectivity using lipases embedded in crude latex from *Carica papaya*. An enantioselectivity E-value E > 200 was achieved using decane as solvent, 50 mM of substrate and 50 mg/mL enzyme/reaction medium. Furthermore, the E-value doubled after purifying latex and removing proteases. 

## 5. Conclusions

As outlined above, Biocatalysis is currently employed in a number of processes and products in diverse fields, and certainly new areas of application will be added. Advances in computational power have enabled the advent of bioinformatics, genomic sequencing and powerful analytic methods in physics, chemistry and molecular biology, which have allowed us to understand the dynamics, *in vivo* and *in vitro*, of biomacromolecules. The convergence of these tools has led to an improved access to more biocatalysts and to a deeper knowledge of them. In the case of enzymes, it has allowed us to explore their behavior, stability, specificity and even to begin to modify their very own nature through protein engineering. Larger availability of biocatalysts with superior qualities has been the main force behind the development of new industrial biocatalytic processes. The continued progress and interest in enzymes, serves as stimulus to make further efforts and ensure a steady success in meeting new synthetic challenges. 

As per the environmental and economic benefits already reaped by the use of Biocatalysis in industry, it is possible to tell that the use of Biocatalysis is only expected to grow. This might be achieved with isolated enzymes but will probably be more successful using genetically engineered organisms where new pathways might be designed. The development of more robust, versatile, efficient enzymes via protein engineering, faster optimization of reaction conditions, microscale processing and better capabilities in design and machining new equipment will open new perspectives for the manufacture of many more products via Biocatalysis. 

Future developments in Biocatalysis for industrial applications should be directed into the expansion of capabilities of current biocatalysts, to obtain new biocatalytic activities able to transform the vast amount of non-natural compounds found in industry and to achieve productivities comparable to current chemical processes. There is no doubt that modern biological tools, such as protein engineering and directed evolution will play an important role in meeting these challenges. As biodiversity remains largely unexplored, developing high-throughput screening methods to discover new efficient enzymes will also impact upon the development of biocatalyzed process for production of biobased chemicals.
